# Distributed K-Means algorithm based on a Spark optimization sample

**DOI:** 10.1371/journal.pone.0308993

**Published:** 2024-12-23

**Authors:** Yongan Feng, Jiapeng Zou, Wanjun Liu, Fu Lv

**Affiliations:** Liaoning Technical University, Huludao, China; TTU: Tan Tao University, VIET NAM

## Abstract

To address the instability and performance issues of the classical K-Means algorithm when dealing with massive datasets, we propose SOSK-Means, an improved K-Means algorithm based on Spark optimization. SOSK-Means incorporates several key modifications to enhance the clustering process.Firstly, a weighted jump-bank approach is introduced to enable efficient random sampling and preclustering. By incorporating weights and jump pointers, this approach improves the quality of initial centers and reduces sensitivity to their selection. Secondly, we utilize a weighted max-min distance with variance to calculate distances, considering both weight and variance information. This enables SOSK-Means to identify clusters that are farther apart and denser, enhancing clustering accuracy. The selection of the best initial centers is performed using the mean square error criterion. This ensures that the initial centers better represent the distribution and structure of the dataset, leading to improved clustering performance. During the iteration process, a novel distance comparison method is employed to reduce computation time, optimizing the overall efficiency of the algorithm. Additionally, SOSK-Means incorporates a Directed Acyclic Graph (DAG) to optimize performance through distributed strategies, leveraging the capabilities of the Spark framework. Experimental results show that SOSK-Means significantly improves computational speed while maintaining high computational accuracy.

## 1 Introduction

Clustering is an unsupervised learning algorithm that can partition multiple classes without prior training. Common clustering algorithms can be classified into the following categories [1]: hierarchical, partitioning, density-based, grid-based, and model-based methods. The K-Means algorithm is one of the partitioning methods and has been widely used in scientific and industrial fields due to its simplicity, low time consumption, and low space complexity. However, prior to its application, we have to determine two parameters: the number of clusters and the initial clustering centers. The two parameters can directly affect the accuracy of the clustering result. The classical K-Means algorithm is especially sensitive to the initial clustering centers; that is, the clustering results change according to the choice of initial centers, and random centers easily cause the algorithm to fall into a local optimum. The direct application of the K-Means method may obtain poor results. In addition, when traditional serial K-Means clustering is used to handle massive data, the performance of the algorithms decreases rapidly because the memory and time can be restricted.

Therefore, for the above two problems in the traditional K-Means algorithm, this paper makes the following improvements based on the research of:

1. We utilize a weighted jump library approach to perform random sampling and preclustering, incorporating the concepts of weights and jump pointers.
2. We employ a weighted max-min distance with variance technique, which considers both the weight and variance information during distance calculation.
3. We determine the best initial centers using the mean square error approach, ensuring that the initial centers accurately represent the distribution and structure of the dataset.
4. A novel distance comparison method is employed to optimize the iterative process and reduce computation time.
5. The algorithm also describes the DAG, which can be used to optimize performance based on a distributed strategy.

The organization of the remaining paper is as follows: Section 2 introduces the improvement of the K-Means algorithm, including related distributed work; Section 3 introduces the basic knowledge of this paper; Section 4 introduces the SOSK-Means algorithm; Section 5 shows a comparison of experiments and analysis; and we conclude the paper in Section 6.

## 2 Related work

Researchers are addressing the sensitivity of the K-Means algorithm to initial centroids and outliers, enhancing algorithmic stability and scalability in processing large datasets. Researchers have developed various methods to address the sensitivity of the K-Means algorithm to initial centroids and outliers. Oliveira et al. [2] proposed two scalable metaheuristic algorithms for clustering large datasets in MapReduce. The first method iteratively enhances k-means clustering using evolutionary operators, while the second method applies evolutionary k-means to the distributed part of the dataset and merges the results. Al-Kababchee et al. [3] proposed an enhanced K-means clustering method using balanced optimization that adjusts the number of clusters and selects attributes for best results. Chen et al. [4] proposed a method that calculates the maximum distance between two data objects to determine initial centroids. Pun et al. [5] suggested using entropy and kurtosis coefficient values to select the distance metric for clustering. Liao et al. [6] combined density-based selection with distance measures. Thamer et al. [7] proposed an enhanced kernel K-means clustering method that effectively tunes the hyperparameters of the kernel function and the number of clusters. Arthur et al. [8] introduced a sampling-based K-Means++ algorithm that prioritizes points further away from the current centroids. Bahmani et al. [9] improved the K-Means++ algorithm with the K-Means|| algorithm suitable for massive data on MapReduce. Cui et al. [10] proposed a weighted and distribution-based merge strategy. Sarch et al. [11] proposed an improved nature-inspired algorithm for penalized regression-based clustering, which enhances its estimation capabilities in genetic analysis and data mining applications. Fahim et al. [12] improved the center allocation process by considering the distance from new and old centroids. Zhao et al. [13] developed a parallel K-Means algorithm based on MapReduce, optimizing network communication. Moertini et al. [14] enhanced the algorithm to handle noise and outliers through data preprocessing. Yin et al. [15] introduced a random sample-based K-Means algorithm on MapReduce. Cai et al. improved centroid selection using sample neighborhood and intra-size variance. Lei et al. [16] proposed an efficient clustering algorithm based on local optimality and graph search. These algorithmic improvements have mostly been implemented on MapReduce, although Spark has also been used for optimization due to its memory computing capabilities. Zakariya et al. [17] proposed an improved nature-inspired algorithm for penalized regression-based clustering that enhances its estimation capabilities in genetic analysis and data mining applications. Kusuma et al. [5] introduced an intelligent K-Means algorithm on Spark that clusters data close to outliers separately, removes objects in the outlier-formed cluster, and iteratively finds new outliers as initial centroids. Kababchee et al. [17] proposed an improved nature-inspired algorithm for penalized regression-based clustering, which enhances its estimation capabilities in genetic analysis and data mining applications. Wang et al. [18] emphasized the flexibility of the Spark-based K-Means algorithm, allowing the selection of different distance functions and computational methods for distance and centroid updates. Lydia et al. [19] demonstrated that Spark-based K-Means outperforms MapReduce in terms of execution time, scheduling delay, acceleration ratio, and resource consumption. Liu et al. [20] developed a parallel K-Means algorithm for massive texts on Spark using resilient distributed datasets (RDDs) for improved computational efficiency. Santhi et al. [21] proposed an optimized K-Means clustering technique incorporating the Bat algorithm and Firefly algorithm to determine optimal initial centroids on Spark. Amal et al. [22] proposed an adaptive firefly optimization algorithm that improves the performance of K-means clustering methods by solving the challenges of determining the number of clusters and the optimal centroids. Sinha et al. [23] addressed the issue of determining the number of clusters and proposed a novel K-Means-based algorithm that dynamically decides the number of clusters based on increasing k values and setting a threshold for optimal cluster generation.

## 3 Basic knowledge

### 3.1 K-Means algorithm

K-Means is one of the partition-based clustering algorithms. For the dataset containing *n* data objects, the K-Means algorithm clusters these objects into *k* clusters. Data objects are assigned to the cluster centres, which are chosen randomly, and at the end of each iteration, the cluster centres are updated until there is no change in the centres.

The pseudocode of the K-Means algorithm is shown in Algorithm 1:

**Algorithm 1** K-Means Clustering

1: **Input**: dataset *D*, the number of clusters *K*, maximum number of iterations *itr*

2: **Output**: *K* clusters and *K* cluster centroids

3: randomly choose *K* objects from *D* as the initial centroids *C*

4: **while** *t* > *itr* **do**

5:  **for** each object *x*_*i*_ in *D* **do**

6:   **for** each centroid *C*_*j*_ in *C* **do**

7:    calculate the distance from *x*_*i*_ to *C*_*j*_

8:   **end for**

9:   assign *x*_*i*_ to the nearest centroid

10:  **end for**

11:  calculate new cluster centres

12: **end while**

### 3.2 Random sampling

Random sampling selects a random sample of size *n* from a set of size *N* (where *n* ≤ *N*) with the goal of ensuring that all data can be selected. Although this method is simple, it may not be suitable for uncertain datasets that require numbering during sampling. However, in data mining, where the overall data is known, the uncertainty of the entire dataset does not affect sampling, leading to better results. Random sampling is particularly suitable for large-scale data processing as a larger sample size can better represent the entire dataset.

### 3.3 Reservoir sampling

Reservoir sampling is a simple and unbiased random sampling technique proposed in the literature [24], which selects without replacement a random sample of size *n* from a set of size *N*. The term “reservoir” defines a storage area for storing potential candidates for the sample. The first step of the reservoir algorithm is to put the first *m* records of the file into a “reservoir”. Then, the record is iterated in sequence to evaluate whether the record replaces the existing record in the reservoir. If it passes the evaluation, a record is randomly selected from the reservoir for replacement. When all records are traversed, a random sample of size *n* can be generated. Reservoir sampling can ensure that each record is drawn with equal probability.

## 4 SOSK-Means algorithm

### 4.1 Concept of the algorithm

The classical K-Means algorithm begins by randomly selecting initial centroid points for clustering. However, this approach is sensitive, as the clustering results are influenced by the initial centroids, and random selection can lead to local optima [1]. Therefore, it is crucial to choose suitable initial centroids for the K-Means algorithm. Sampling, a commonly used statistical method, has been introduced into clustering. Arthur et al. [8] selects one sample at a time based on probabilities, with objects that are farther away having a higher probability of being chosen. Other references select multiple samples at once, considering the probability and incorporating the mutual distance between data objects. However, this method’s limitation is that the selection of the next sample depends on the previously selected sample, and only distance is considered for selecting outliers.

In a method that considers overall features and determines the appropriate number of samples, initial centroids are selected through pre-clustering. Yin et al. [15] shows that increasing the number of samples can significantly improve the clustering results’ quality. If the number of pre-clusters *k*′ ≫ *k*, it increases the likelihood of obtaining the global optimum.

In this paper, the SOSK-Means algorithm utilizes the weighted jump reservoir method to perform random sampling *i* times on the dataset, obtaining *n*′ samples for pre-clustering, and forming *k*′ clusters and *k*′ initial cluster centroids at each sampling. This approach considers that intra-cluster data distributions, cluster sizes, and inter-cluster distances are different. The algorithm calculates the weighted intra-cluster variance by evaluating the radius and variance of each cluster, where variance reflects the degree of dispersion of data objects from the centroid in the cluster. The SOSK-Means algorithm employs the weighted max-min distance with variance to select *k* clusters, reducing the chance of selecting sparse clusters with outliers. The selected *k* clusters have dense intra-cluster data and well-separated inter-cluster data. By calculating the centroid values of the *k* clusters, *ik* centroid values can be obtained. The best initial centroid is then selected based on the mean square error, representing the original data’s clustering centroids. During the iteration, a novel distance comparison method is used to reduce computation time.

### 4.2 Weighted jump researvoir sampling

From the previous section, reservoir sampling can ensure the randomness of sampling when dealing with large data of unknown size. Since each data point has the same probability of being extracted, this method can be regarded as uniform random sampling. However, if the distribution of the dataset is extremely uneven, the sample taken may not be sufficient to represent the entire dataset. If the sample is selected by iterating all the data, it increases the computational cost.

Therefore, based on the literature [25], this paper improves reservoir sampling and proposes weighted jump reservoir sampling. *v*_*i*_ is assumed to be a data point, and the weight of *v*_*i*_ is *w*_*i*_ (*w*_*i*_ > 0). The random function random(*L*, *H*) generates a uniform random number in (*L*, *H*). Given a random variable *X*, *F*_*X*_(*x*) is its distribution function.

**Definition 1**: Assume *U*_1_ and *U*_2_ are random variables that obey a uniform distribution in [0, 1], $X_{1}={\left(U_{1}\right)}^{1/w_{1}}$, $X_{2}={\left(U_{2}\right)}^{1/w_{2}}$, *w*_1_, *w*_2_ > 0, then:

$$\begin{array}{c} P\left(X_{1}\leq X_{2}\right)=\frac{w_{2}}{w_{1}+w_{2}} \end{array}$$ (1)

Therefore, the probability that the data point is selected for sampling is proportional to its weight *w*_*i*_, and the probability of each data point being selected is not known before sampling. If the weight of a data point is greater than that of other data points, the closer the value of *X*_*i*_ is to 1, the more likely the data are to be saved. In the following, *X*_*i*_ represents the feature value *k*_*i*_.

**Definition 2**: It can be seen from the mathematical induction that for *α* ∈ [0, 1], *w*_*i*_ > 0, $X_{i}={\left(U_{i}\right)}^{1/w_{i}}$, *U*_*i*_ ∈ *U*(0, 1), then:

$$\begin{array}{c} F_{X_{i}}\left(\alpha \right)=P\left\{X_{i}\leq \alpha \right\}=P\left\{U_{i}^{\left(1/w_{i}\right)}\leq \alpha \right\}=P\left\{U_{i}\leq \alpha^{w_{i}}\right\} \end{array}$$ (2)

The smallest key in *R* is the current threshold *T*. The following random variable *X*_*w*_ can be used to generate the appropriate exponential “jumps”:

$$\begin{array}{c} X_{w}=\frac{\text{ln}(\text{random}(0,1))}{\text{ln}(T)} \end{array}$$ (3)

Let *W*_*i*,*j*_ = *w*_*i*_ + *w*_*i*+1_ + … + *w*_*j*_(*i* ≤ *j*); when *X*_*w*_ satisfies the following formula, the data for *v*_*i*_ is obtained:

$$\begin{array}{c} W_{c,i-1}<X_{w}<W_{c,i} \end{array}$$ (4)

The probability of jumping to read *v*_*i*_ from Eq (2) is as follows:

$$\begin{array}{c} \begin{array}{cc} P(W_{c,i-1}<X_{w}<W_{c,i}) & =P(W_{c,i-1}<\frac{\text{ln}(U)}{\text{ln}(T)}<W_{c,i})\\ & =P(T^{W_{c,i}}\leq U<T^{W_{c,i-1}})\\ & =F_{U}(T^{W_{c,i-1}})-F_{U}(T^{W_{c,i}})\\ & =P(U\leq T^{W_{c,i-1}})-P(U\leq T^{W_{c,i}})\\ & =T^{W_{c,i-1}}-T^{W_{c,i}} \end{array} \end{array}$$ (5)

The size of *v*_*i*_’s eigenvalues, *k*_*i*_ and *T* are compared to decide whether to put it in the pool for replacement. Then, the probability of entering the pool by Eq (2) is as follows:

$$\begin{array}{c} P(U_{i}^{\frac{1}{w_{i}}}>T)=1-T^{w_{i}} \end{array}$$ (6)

**Definition 3**: Assume that the algorithm has processed *c* − 1 items (*c* > *m*). *T* is the current threshold in the reservoir. The next item to be examined is *V*_*c*_. According to Eqs (2) and (4), for each *i* = *c*, *c* + 1, …, *n*, the probability *P*_next_(*V*_*i*_) that *V*_*i*_ is the next item that enters the reservoir is:

$$\begin{array}{c} \begin{array}{cc} P_{\text{next}}\left(V_{i}\right) & =P\{W_{c,i-1}<X_{w}⩽W_{c,i}\}\cdot P\{U_{i}^{\frac{1}{w_{i}}}>T\}\\ & =(T^{W_{c,i-1}}-T^{W_{c,i}})\cdot (1-T^{W_{i}})\\ & =T^{W_{c,i-1}}-2T^{W_{c,i}}+T^{W_{c,i}+w_{i}} \end{array} \end{array}$$ (7)

The two main operations of weighted jump reservoir sampling are generating weights for data and reading data by jumping and using a reservoir of size *m* to store the final sample candidates. The comparison of feature values can ensure that the data with greater weight are more likely to be retained. Jumping reduces the number of generated random variables. All operations can be completed by one iteration of the data set. Since the computational cost of generating a large number of random variables may be high, weighted jumping reservoir sampling improves the complexity of classical reservoir sampling.

The pseudocode of weighted jump reservoir sampling is as Algorithmic 2.

**Algorithm 2** Weighted Jump Reservoir Sampling

1: **Input**: A population *V* of *n* weighted data

2: **Output**: A reservoir *R* of size *m*

3: Insert the first *m* items of *V* into *R*

4: **for** each item *x*_*i*_ ∈ *R* **do**

5:  Calculate a key $k_{i}=u_{i}^{1/w_{i}}$, where *u*_*i*_ = random(0, 1)

6: **end for**

7: The smallest key *k*_*i*_ in *R* is the current threshold *T*

8: **repeat**

9:  Generate *X*_*w*_ according to Eq (3)

10:  Jump from the current data *V*_*c*_ to *V*_*i*_ and satisfy inequality (4); then, make *V*_*i*_ become *V*_*c*_

11:  Let $t_{w}=T^{w_{i}}$, *r* = random(*t*_*w*_, 1), calculate $k_{i}=r^{1/w_{i}}$

12: **until** the data set is processed

13: **if** *k*_*i*_ > *T* **then**

14:  Replace the data item with the minimum key *T* in *R* with item *v*

15:  Calculate the new *T* in *R*

16: **end if**

17: Calculate the new T in R

### 4.3 Sample-based initial cluster centroid selection scheme

The original dataset is assumed to be {*x*_1_, *x*_2_, …, *x*_*n*_}, where *n* is the size of the dataset, and each data point has *w*-dimensional features, i.e., *x*_*i*_ = (*x*_*i*1_, *x*_*i*2_, …, *x*_*iw*_). The dataset is divided into *k* clusters, and the centres of the data clusters are *c*_1_, *c*_2_, …, *c*_*k*_.

After sampling the weighted jump reservoir to obtain the sample dataset, pre-clustering the dataset is equivalent to performing a cluster. The pre-clustering number *k*′ is much larger than the real clustering number *k* because the K-Means algorithm is a stochastic hill-climbing algorithm on the logarithm likelihood function space, making *k*′ ≫ *k* similar to developing more climbing paths, which increases the possibility of reaching the global optimum.

The literature [16] recommends that the value of *k*′ is $\frac{n}{\mu_{\text{min}}}$, where *μ*_min_ is the amount of data contained in the smallest cluster, and *n* is the overall amount of data. However, sometimes it is difficult to know the value of *n* for a large amount of data, so the algorithm in this paper automatically counts *n* values and then uses the value of *n* to calculate *k*′.

**Definition 4**: The selection of the pre-clustering number *k*′ is:

$$\begin{array}{c} k^{\prime }=\text{min}\left(\alpha \cdot k,n^{5/8}\right),\;1\leq \alpha \leq 10s \end{array}$$ (8)

where *n*/*k* is replaced by *μ*_min_ because if the value of *n* cannot be determined, the value cannot be accurately obtained.

At the same time, to ensure that *k*′ centroids can be clustered in the algorithm, the number of samples should be greater than *k*′; otherwise, clustering cannot be performed. Therefore, when the number of samples is larger than max(*α* ⋅ *k*, *n*^5/8^), it is obvious that max(*α* ⋅ *k*, *n*^5/8^) ≥ min(*α* ⋅ *k*, *n*^5/8^).

### 4.4 Selecting initial cluster centroids

k’ clusters are formed by pre-clustering the sample, k clusters are selected by the maximum and minimum distances with intra-cluster variance, and then the initial centre is obtained.

(1) Cluster Radius

The cluster radius is the maximum distance between all the points and the centroid, and its value is:

$$\begin{array}{c} \gamma_{k}=\text{max}\left\{d\left(x_{i},c_{k}\right)\right\} \end{array}$$ (9)

where *x*_*i*_ is the object in cluster *k*, and *d*(*x*_*i*_, *c*_*k*_) is the Euclidean distance between the data point *x*_*i*_ and the centroid *c*_*k*_. (2) Intra-cluster Variance

The variance is used to measure the discrete degree between data and expectation values (i.e., the mean). The intra-cluster variance is calculated as follows:

$$\begin{array}{c} s_{k}=\frac{\sum_{x_{i}\in c_{k}}{\left[d\left(x_{i},c_{k}\right)-M_{k}\right]}^{2}}{|C_{k}|-1} \end{array}$$ (10)

where *M*_*k*_ is the average distance of object *x*_*i*_ to centroid *c*_*k*_, |*C*_*k*_| is the number of data points in cluster *k*, and the intra-cluster variance reflects the degree of deviation of clusters *x*_*i*_ and *M*_*k*_. If the intra-cluster variance is smaller, the intra-cluster objects in the cluster are denser.

After obtaining the radius and variance of each cluster, the variance within the cluster is:

$$\begin{array}{c} s_{k}^{*}=\frac{r_{k}-\text{min}\left(R\right)}{\text{max}(R)-\text{min}(R)}\cdot s_{k} \end{array}$$ (11)

where *R* is the set of all cluster radii, and the fraction in Eq (11) represents the normalization of *r*_*k*_ to a number in [0, 1].

(3) Weighted Max-Min Distance with Variance

After pre-clustering into *k*′ clusters, the cluster centres constitute the candidate set *T* = {*t*_1_, *t*_2_, …, *t*_*k*′_} of the initial cluster centres, and the final set of initial cluster centroids is *C* = {*c*_1_, *c*_2_, …, *c*_*k*_}. For the candidate *t*_*k*_ in *T*, the max-min distance with variance is:

$$\begin{array}{c} d_{k}^{*}=\text{min}\left\{d\left(c_{i},t_{k}\right)\right\}\cdot \frac{1}{s_{k}^{*}},\;c_{i}\in C \end{array}$$ (12)

where min{*d*(*c*_*i*_, *t*_*k*_)} is the minimum distance between the candidate *t*_*k*_ and the set *C* of selected initial centroids.

The larger min{*d*(*c*_*i*_, *t*_*k*_)} is, the greater the distance between the candidate and the set of selected initial centroids, the smaller the weighted intra-cluster variance $s_{k}^{*}$ of cluster *k*, and the denser the data distribution. When the value of the weighted max-min distance $d_{k}^{*}$ is larger, it means that intra-cluster data are dense and inter-cluster data are far apart, and the cluster centroid is used as the next initial cluster centroid.

Therefore, the steps to select the initial cluster centre after pre-clustering are as follows:

1. Randomly choose a candidate *t*_*a*_ from *T* as the first initial clustering centroid *c*_1_, *c*_1_ = *t*_*a*_, and delete *t*_*a*_ from *T*.
2. Choose a candidate *t*_*b*_ as the next initial clustering centroid *c*_2_, *c*_2_ = *t*_*b*_, *t*_*b*_ satisfies $d_{b}^{*}=\text{max}\left(d_{k}^{*}\right)$, *t*_*k*_ ∈ *T*, and delete *t*_*b*_ from *T*.
3. Repeat Step 2 until a total of *k* centres are chosen from the remaining candidates in *T*.

In the K-Means algorithm, the Euclidean distance is used to calculate the distance between data, and the data are divided into clusters. In the execution of the algorithm, it is only necessary to calculate which centre is closest to a certain point, and it is not necessary to calculate the exact Euclidean distance value from each data point to each centroid, so a quick comparison method to calculate distance is used in this paper.

**Definition 5**: A distance formula *newDist* is as follows:

$$\begin{array}{c} \text{newDist}=(\sqrt{\sum_{i=1}^{w}c_{1i}^{2}}-\sqrt{\sum_{i=1}^{w}x_{1i}^{2}}{)}^{2} \end{array}$$ (13)

The Euclidean distance *euclDist* of the K-Means algorithm can be calculated by the following equation:

$$\begin{array}{c} \text{euclDist}=\sum_{i=1}^{w}{\left(m_{1i}-x_{1i}\right)}^{2} \end{array}$$ (14)

where *w* is the number of features. It is assumed that the centre point *c* is (*c*_11_, *c*_12_, …, *c*_1*w*_) and the data point *x* is (*x*_11_, *x*_12_, …, *x*_1*w*_). Comparing the two distance formulas, it is easy to show that *newDist* ≤ *euclDist*.

Comparing each data point and the centre point distance, the minimum distance obtained previously by *bestDist* and *newDist* can be obtained by the L2 norm of *m* and *x*. If *newDist* > *bestDist*, then *euclDist* > *bestDist*, then there is no need to calculate *euclDist*, which saves much calculation work. If *newDist* < *bestDist*, then calculate the Euclidean distance *euclDist*, compare its size with *bestDist*, and then directly use the L2 norm of *m* and *x* obtained when calculating *newDist*.

### 4.5 Algorithm distributed strategy

It is found in the literature that traditional clustering algorithms become ineffective in clustering large datasets [26]. MapReduce can solve this problem, but MapReduce is sensitive to iterative calculations. Each round of jobs needs to read data from the disk and write the results back to the magnetic disc file system, which greatly increases system overhead such as I/O, and iterative calculations often occur. Therefore, the K-Means algorithm needs to be optimized for distributed computing. Spark is a new generation of large-scale data processing and computing frameworks. In terms of computing efficiency, its memory-based execution is much faster than MapReduce, which reads and writes to disk multiple times. Lydia et al. [27] pointed out that experiments prove that when the data are in memory, the execution speed of Spark is 100 times faster than MapReduce, and the speed of accessing data from disk is also 10 times faster.

According to the sample dataset obtained by sampling in Section 4.2, the subsequent processing of the SOSK-Means algorithm only processes the sample dataset instead of all the data.

**(a) pre-clustering**

Put the RDD samples in, whitch number is k’. The progress will output an array of pre-clustering results preResults. The step is as folows:

**Step 1**: Sort the RDD samples in descending order, select the first k’ data points as the initial cluster centroids of the pre-clustering, and then broadcast the k’ centroids to all worker nodes.
**Step 2**: Define the internal function merge, which is used to accumulate the value of two key-value sequences with the same key.
**Step 3**: Perform the mapPartitions operation on the RDD example, calculate which cluster the data in each partition belongs to, and generate a key-value sequence.
**Step 4**: Perform a reduceByKey operation on each key-value sequence to summarize, use the merge function to merge the values of the same key, and calculate the global data attributes of each cluster.
**Step 5**: Calculate the sum of squares of the difference between the data in each cluster and the mean sqSums(j) and obtain key-value pairs of the form (j, sqSums(j)).
**Step 6**: Combine the same key j to obtain the global key-value array tempList1.
**Step 7**: According to tempList and tempList1, the key-value array preResults is generated to obtain k’ clusters.

**(b) selecting cluster centroids**

Input the RDD sample and Pre-clustering results preResults, then the array of initial cluster centroids will be output.

**Step 1**: Add the cluster centroid of the cluster with the smallest variance in preResults to centersList as the first initial centroid and delete the centre from preCenters.
**Step 2**: Calculate the weighted variance of each cluster in preResults.
**Step 3**: Do follow steps until the number of elements in centersList is more than k:
  (1) Perform a foreach operation on preResults and calculate the weighted max-min distance from the centre of each cluster to the centre of centersList.
  (2) Select the cluster centre corresponding to the largest distance to add to centersList and delete it from preResults.
**Step 4**: Calculate the error sum squares of the centroid of the group to the RDD sample.

**(c) parallel K-Means clustering**

This progress need input the dataset RDD, maximum number of iterations iter, threshold and the initial centroid array centerList. The updated centre array centerList will be output.

**Step 1**: Broadcast centerList to Worker node.
**Step 2**: Do follow step until the number of iterations is less than or equal to iter or the cluster centre change value is less than threshold:
  (1) Perform the mapPartitions operation on the RDD input, compare the distance from the centroid to the data in each partition, record the index of the centroid to which it belongs, and form a key-value sequence.
  (2) Perform the reduceByKey operation on the key-value sequence, merge the key-value sequence, remove the farthest and nearest data in each key, calculate the new centre point and update the centerList.

### 4.6 Analysis of the processing time

Assuming that the number of rows of the original dataset is *n*_0_, the number of sampling times is *m*, *n*_*s*_ rows of data are extracted each time, and the feature number of each row is *C*. *F*_1_ shuffle operations are generated for *n*_0_, and *F*_2_ shuffle operations are generated for *n*_*s*_. The number of cluster nodes is *S*, the communication bandwidth between the machines in the cluster is *B*, the *i*-th machine is *S*_*i*_, the total time for processing data is *T*(*S*_*i*_), and other time is *t*.

Assuming that the data of each machine are shuffled to other nodes and the amount of data remains unchanged, the total time spent *T* is:

$$\begin{array}{c} T=\frac{\left(S-1\right)\times \left(n_{0}\times F_{1}+m\times n_{s}\times F_{2}\right)\times C}{B}+\text{max}\left\{T\left(s_{i}\right),1\leq i\leq S\right\}+\varepsilon \end{array}$$ (15)

From the above Eq (15), the total time is mainly determined by the amount of data, the number of samples, the number of shuffles, and the execution efficiency of each machine, where *n*_*s*_ is determined by *n*_0_. When the amount of data is constant and *m* is determined, reducing the number of shuffles and optimizing the time overhead of shuffles are the keys to solving the algorithm performance bottleneck.

### 4.7 Algorithm performance optimization

**Fig 1:**
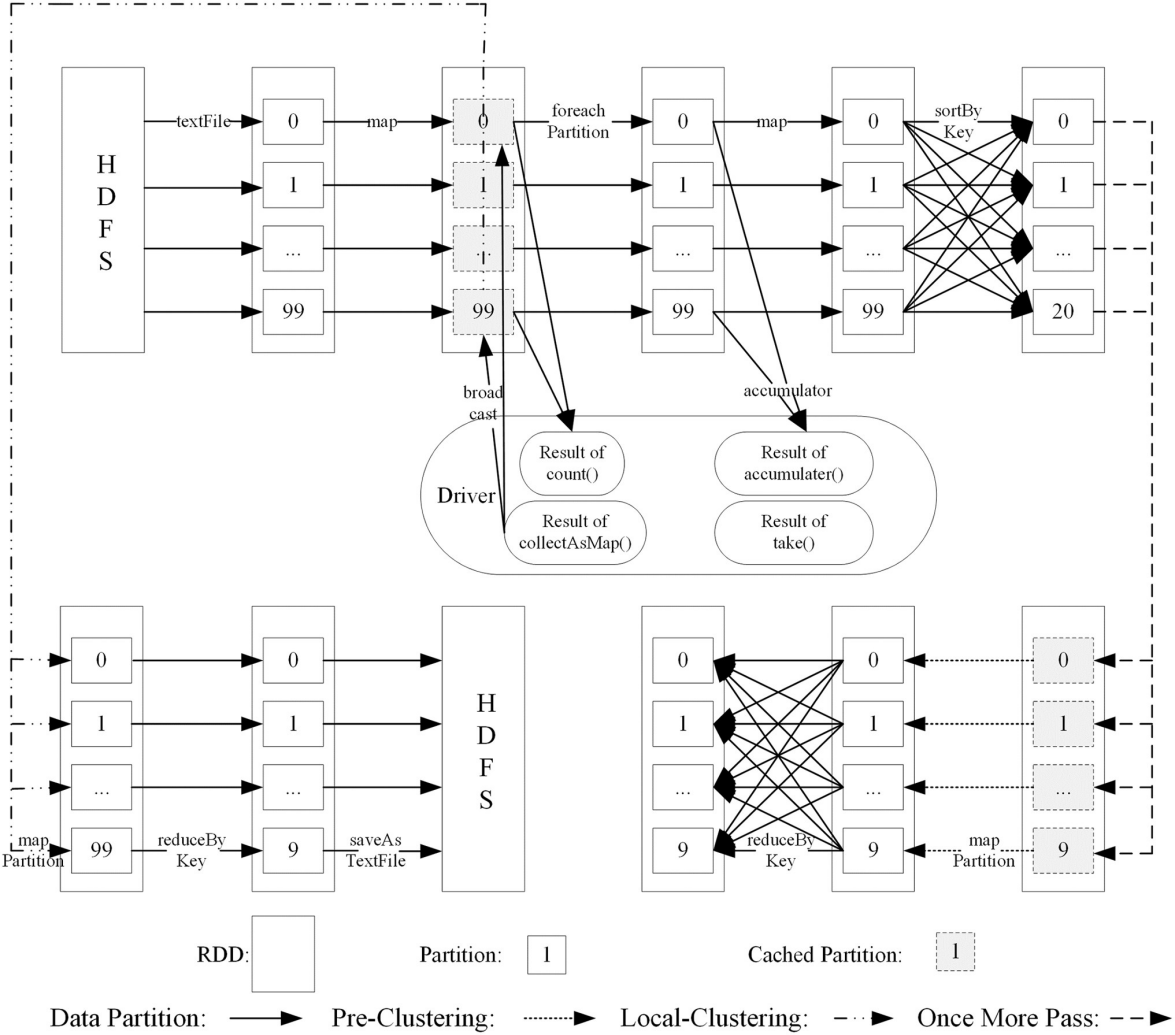
Logic DAG of the soak-Means.

According to the theoretical analysis in the previous section, shuffling is the most performance-consuming part of the algorithm in Spark because this part contains a large number of disk IOs, serialization, network transmission, and other operations. If it is not properly tuned, the execution speed of the algorithm is very slow. Therefore, it is optimized according to the logical DAG diagram of the SOSK-Means algorithm. As shown in Fig 1, the application of the SOSK-Means algorithm generates at least four jobs:

- **job_0**: Triggered by the count operator, there is only one stage, used to read in the statistics of the data set.
- **job_1**: Triggered by the take operator and divided into two stages: use the accumulator variable to summarize the calculation results and use the shuffle operation to extract sample data from the original data set.
- **job_i**: Triggered by the pre-clustering collectAsMap operator, where *i* ≥ 1, and the value of *i* depends on the number of samples. Two stages are generated: the broadcast operation of the pre-clustering centre and the shuffle operation. Used for pre-clustering to select the initial centre.
- **job_j**: Triggered by the collectAsMap operator of iterative clustering, where *j* ≥ 1, and the value of *j* depends on the number of iterations. Contains two stages: the broadcast operation of the optimal initial cluster centre and the shuffle operation. According to the algorithm’s DAG graph, it is optimized in the following aspects:

**(1) Shuffle operator is avoided**

No shuffle is generated in job_0. This job reads the original data set to form an RDD. Since this RDD is used multiple times, it is cached and persists in the memory to avoid cumbersome calculation from the source every time the operator is executed. Because the RDD data are directly extracted from the memory and then the shuffle operation is performed, the cost of reading and writing to the disk is reduced, and it is suitable for the iterative calculation of the algorithm.

**(2) Shuffle operator is used to process partition**

In job_1, the foreachPartition operator calculates the sum of all samples in each partition with one call. Compared with the one-time call of the foreach operator to process a piece of data, it is of great help to the performance improvement. Similarly, the mapPartition operator that performs local clustering in each partition in job_i improves the performance similarly to foreachPartition, instead of the normal map operator, calling to process one partition at a time.

**(3) Shuffle operators are used for pre-aggregation**

Pre-aggregation means that when the task of the current stage executes shuffle write, each node performs an aggregation operation on the same key locally. Since multiple identical keys are merged, each node has only one key locally, and then the next one. When the stage task executes shuffle read, when other nodes pull the same key on all nodes, it greatly reduces the amount of data that needs to be pulled, thereby reducing disk IO and network transmission overhead.

The reduceByKey operator is used in both job_i and job_j to replace the groupByKey operator. Because the reduceByKey operator pre-aggregates the same key locally according to a user-defined function, the groupByKey operator does not pre-aggregate, and the performance is relatively poor compared to that of reduceByKey.

**(4) Broadcast external variables**

Both the pre-clustering and iterative clustering of the algorithm use the external variables that save the initial centre. By default, Spark replicates [replicate] multiple copies of this variable and transfer them to each task in Executor via the network. Especially in pre-clustering, the number of initial centres is much larger than in iterative clustering. If this variable is large, a large number of copies is transmitted in the network, occupying the memory of Executor in each node and affecting performance.

Therefore, for the above situation, the variable is broadcast. The broadcast variable ensures that only one copy of the variable resides in the memory of each Executor, and the task shares the copy, which not only reduces the number of copies and Executor memory overhead but also avoids variables. The load generated by multiple transmissions between nodes improves execution efficiency.

**(5) Merge temporary files**

In the shuffle write process, by default, the task of the current stage creates a temporary disk file for each task of the next stage. When it is the turn of the next batch of tasks to execute, a new temporary file is created, so many temporary files are generated, which affects performance.

The consolidation mechanism is turned on for this. When Executor executes a batch of tasks and then executes the next batch of tasks, the next batch of tasks does not create new files but reuse the files created by the previous batch of tasks and write the data into an existing temporary file. In this way, files created by multiple tasks can be merged to a certain extent, thereby greatly reducing the number of temporary files and improving shuffle write performance.

**(6) Dual aggregation**

In clustering, if the amount of data of individual clusters is larger than that of other clusters, that is, the amount of data corresponding to individual keys in the program is too large, when shuffle operators such as reduceByKey are executed, data skew occurs and task execution is particularly slow. During shuffle reading, individual tasks are allocated a larger amount of data, the execution time is slower than other tasks, and the running time of the entire spark job is determined by the slowest task.

In this regard, a dual aggregation solution is adopted to solve this problem. The first time is partial aggregation. After finding the key that caused the data skew, a random number prefix is added to each key. At this time, the same key becomes multiple different keys. Then, aggregation operations are performed on the prefixed data, and partial aggregation is performed so that a large amount of data originally processed by one task can be distributed to multiple tasks for processing. As a result of partial aggregation, multiple different keys become a small number of different keys, but the corresponding data have been aggregated once. Finally, the prefix of each key are removed and another aggregation is performed to obtain the global aggregation result, thereby solving the problem of excessive data processing by a single task.

**(7) Kryo is used for serialization**

When shuffling and caching the RDD above and using broadcast variables, the data need to be serialized before they can be stored. Spark’s default serialization speed is slow, and the serialized data occupy a large amount of memory. Therefore, the Kryo serialization library is set in the configuration file for final optimization to ensure high serialization efficiency.

## 5 Experiment and analysis

### 5.1 Experimental environment

The Spark cluster consists of 1 master node and 8 slave nodes in the Linux system environment. Each node of the Spark cluster runs the CentOS 6.5 operating system, equipped with dual Intel Core (TM) CPUs, 4GB of RAM, and a 500GB hard disk. The cluster is configured with Hadoop 2.6.4, Spark 3.2.3, Scala 2.10.5, and JDK 1.8.5.2.

### 5.2 Description of the datasets

To validate the performance of the proposed algorithm, this paper selects several commonly used synthetic datasets for clustering analysis as well as some real-world large datasets, as detailed below:

The Iris flower (Iris), banknote (Bank), seeds, Wifi_Localization (Wifi), Planning Relax (Plrx), and wine datasets were selected from the UCI Machine Learning Repository. Additionally, the R15 and D31 datasets were obtained from the University of Eastern Finland.

Regarding the real-world datasets, we selected the credit card fraud detection dataset (CCFD) [28] and the KDD Cup 1999 Data (KDDc99) [29]. The CCFD dataset contains 284,807 instances with 30 features, including Time, Amount, and 28 anonymized PCA-transformed features (V1 to V28), and is highly imbalanced with only 0.172% fraudulent transactions. The KDDc99 dataset includes 4,898,431 instances with 41 features categorized into basic, content, and traffic features, suitable for clustering and classification tasks in network intrusion detection, with multiple classes representing different types of intrusions and normal connections.

All the datasets along with their details are shown in Table 1.

**Table 1:** Database details.

Dataset	Number of Instances	Number of Features	Number of Classes
R15	600	2	15
D31	3100	2	31
Iris	150	4	3
Bank	1372	4	2
Seeds	210	7	3
Wifi	2000	7	4
Plrx	182	12	2
Wine	178	13	3
CCFD	284807	29	2
KDDc99	48984317	41	5

### 5.3 Experimental design

We conducted a comparative experiments and an performance experiment. We compared SOSK-Means with the following baseline methods:

- **K-means**: Spark MLlib implements a scalable and efficient K-means clustering algorithm, utilizing distributed memory computing to handle large-scale datasets
- **Bisecting K-means**: Spark MLlib provides a Bisecting K-means clustering algorithm, leveraging hierarchical clustering and distributed memory computing for large-scale data processing.
- **EOAK-means** [3]: EOAK-means enhances the traditional K-means clustering algorithm by integrating the Equilibrium Optimization Algorithm (EOA) to select the optimal number of clusters dynamically and improve clustering quality.
- **K-Plus Anticlustering** [30]: This method extends k-means by considering distribution moments (means, variance, higher-order moments) to maximize between-group similarity. It partitions elements into disjoint groups to maximize between-group similarity and within-group heterogeneity, reversing the logic of cluster analysis.
- **LBKC** [31]: This method utilizes the lower bound of Euclidean distance to safely avoid a large number of unnecessary distance calculations, thereby accelerating the k-means process.

The comparative experiment‘s performance was evaluated using 5 standard metrics: accuracy, recall, jaccard index, rand index, and F-score.

### 5.4 Experiment of the clustering effect

For the accuracy, recall, Jaccard index, Rand index, F1-score and time values of each algorithms on each dataset, the results are shown in Tables 2 to 7.

**Table 2:** Comparison of the ACCURACY of the datasets between each algorithm.

Algorithm	R15	D31	Iris	Bank	Seeds	Wifi	Plrx	Wine	CCFD	KDDc99
K-Means	0.9827	0.9057	**0.8262**	0.7831	0.9012	0.9046	0.5943	0.7638	0.8636	0.8668
Bisecting K-means	**0.9875**	0.9060	0.8225	0.7855	0.9102	**0.9060**	0.5955	**0.7645**	0.8654	0.8699
EOAK-means	0.9855	0.9025	0.8185	0.7820	0.9087	0.9030	0.5930	0.7625	0.8636	0.8666
K-Plus Anticlustering	0.9834	0.9045	0.8206	**0.7860**	**0.9107**	0.9036	**0.5956**	0.7634	0.8688	0.8697
LBKC	0.9865	**0.9062**	0.8199	0.7828	0.9099	0.9028	0.5948	0.7635	0.8674	0.8658
SOSK-Means	0.9866	0.9037	0.8207	0.7831	0.9094	0.9039	0.5940	0.7638	**0.8708**	**0.8712**

**Table 3:** Comparison of the RECALL of the datasets between each algorithm.

Algorithm	R15	D31	Iris	Bank	Seeds	Wifi	Plrx	Wine	CCFD	KDDc99
K-Means	0.9814	**0.9171**	**0.8504**	**0.8562**	0.9154	**0.9193**	0.5078	0.7544	0.8838	0.8846
Bisecting K-means	0.9872	0.9055	0.8230	0.7850	0.9100	0.9055	0.5060	**0.7650**	0.8856	0.8864
EOAK-means	0.9860	0.9020	0.8180	0.7810	0.9085	0.9030	0.5020	0.7620	0.8887	0.8868
K-Plus Anticlustering	0.9879	0.9088	0.8485	0.8436	0.9135	0.9059	0.5030	0.7633	0.8874	0.8884
LBKC	0.9846	0.9125	0.8485	0.8220	**0.9177**	0.9132	0.5033	0.7625	0.8890	0.8896
SOSK-Means	**0.9880**	0.9151	**0.8504**	**0.8562**	0.9138	0.9192	**0.5094**	0.7544	**0.8899**	**0.8920**

**Table 4:** Comparison of the JACCARD INDEX of the datasets between each algorithm.

Algorithm	R15	D31	Iris	Bank	Seeds	Wifi	Plrx	Wine	CCFD	KDDc99
K-Means	0.9764	**0.8469**	0.7607	**0.6921**	0.8303	**0.8670**	0.3769	**0.6194**	0.7562	0.7413
Bisecting K-means	**0.9775**	0.8410	**0.7625**	0.6920	0.8310	0.8650	0.3771	0.6180	0.7438	0.7510
EOAK-means	0.9762	0.8420	0.7589	0.6910	0.8331	0.8653	0.3756	0.6123	0.7531	0.7425
K-Plus Anticlustering	0.9751	0.8425	0.7605	0.6919	0.8342	0.8661	0.3730	0.6125	0.7511	0.7413
LBKC	0.9744	0.8425	0.7605	0.6911	0.8337	0.8630	0.3741	0.6113	0.7577	0.7587
SOSK-Means	0.9750	0.8424	0.7606	**0.6921**	**0.8344**	0.8665	**0.3780**	**0.6194**	**0.7639**	**0.7599**

**Table 5:** Comparison of the RAND INDEX of the datasets between each algorithm.

Algorithm	R15	D31	Iris	Bank	Seeds	Wifi	Plrx	Wine	CCFD	KDDc99
K-Means	**0.9984**	**0.9919**	0.8866	**0.8073**	**0.9437**	**0.9543**	0.5051	**0.8419**	0.8326	0.8218
Bisecting K-means	0.9875	0.9850	0.8891	0.7950	0.9340	0.9540	0.5010	0.8350	0.8357	0.8210
EOAK-means	0.9815	0.9915	0.8785	0.8020	0.9385	0.9530	0.5030	0.8325	0.8298	0.8227
K-Plus Anticlustering	0.9839	0.9825	0.8834	0.7959	0.9387	0.9533	0.5028	0.8405	0.8352	0.8211
LBKC	0.9921	0.9895	0.8885	0.7951	0.9364	0.9495	0.5050	0.8368	0.8339	0.8246
SOSK-Means	0.9982	0.9916	**0.8897**	**0.8073**	0.9400	**0.9543**	**0.5059**	**0.8419**	**0.8398**	**0.8287**

**Table 6:** Comparison of the F1-score of the datasets between each algorithm.

Algorithm	R15	D31	Iris	Bank	Seeds	Wifi	Plrx	Wine	CCFD	KDDc99
K-Means	0.9821	**0.9114**	**0.8381**	**0.8155**	0.9083	**0.9119**	0.5473	0.7579	0.8720	0.8750
Bisecting K-means	**0.9873**	0.9058	0.8227	0.7852	0.9101	0.9058	0.5500	**0.7648**	0.8755	0.8781
EOAK-means	0.9857	0.9022	0.8183	0.7815	0.9086	0.9030	0.5435	0.7622	0.8761	0.8767
K-Plus Anticlustering	0.9856	0.9066	0.8334	0.8092	0.9121	0.9048	0.5443	0.7634	0.8781	0.8790
LBKC	0.9855	0.9093	0.8333	0.8014	**0.9138**	0.9079	0.5441	0.7626	0.8782	0.8767
SOSK-Means	**0.9873**	0.9092	0.8357	**0.8155**	0.9116	0.9116	**0.5507**	0.7581	**0.8803**	**0.8816**

**Table 7:** Comparison of the TIME of the datasets between each algorithm(unit:s).

Algorithm	R15	D31	Iris	Bank	Seeds	Wifi	Plrx	Wine	CCFD	KDDc99
K-Means	0.754	3.006	0.186	1.643	0.195	1.895	0.145	0.154	1140.999	5700.562
Bisecting K-means	0.752	3.003	0.185	1.643	0.194	1.894	0.143	0.153	1138.962	5695.378
EOAK-means	0.753	3.004	0.186	1.644	0.194	1.894	0.145	0.153	1137.659	5693.372
K-Plus Anticlustering	0.750	3.005	0.183	1.642	0.193	1.893	0.144	0.154	1135.629	5692.578
LBKC	0.751	3.004	0.183	1.640	0.190	1.89	0.143	0.153	1133.529	5691.957
SOSK-Means	**0.682**	**2.545**	**0.151**	**1.398**	**0.073**	**1.712**	**0.085**	**0.099**	**1021.399**	**5052.753**

According to the experimental results, the clustering indices of the SOSK-Means algorithm are similar to those of the baseline algorithm, indicating that the algorithm achieves high computational accuracy while maintaining high computational speed. Overall, the SOSK-Means algorithm performs well on most datasets. However, due to the random generation of multiple sets of initial centers, the indeterminable randomness of the SOSK-Means algorithm results in average clustering performance.

### 5.5 Performance experiment

To measure the calculation speed of the algorithm, the SOSK-Mean algorithm uses 5 million rows (689 M), 10 million rows (1.34 G), 15 million rows (2.01 G), 20 million rows (2.69 G), 25 million rows (3.36 G), and 30 million rows (4.03 G) of artificially synthesized data sets. The results are compared with those of the K-Means algorithm.

**Fig 2:**
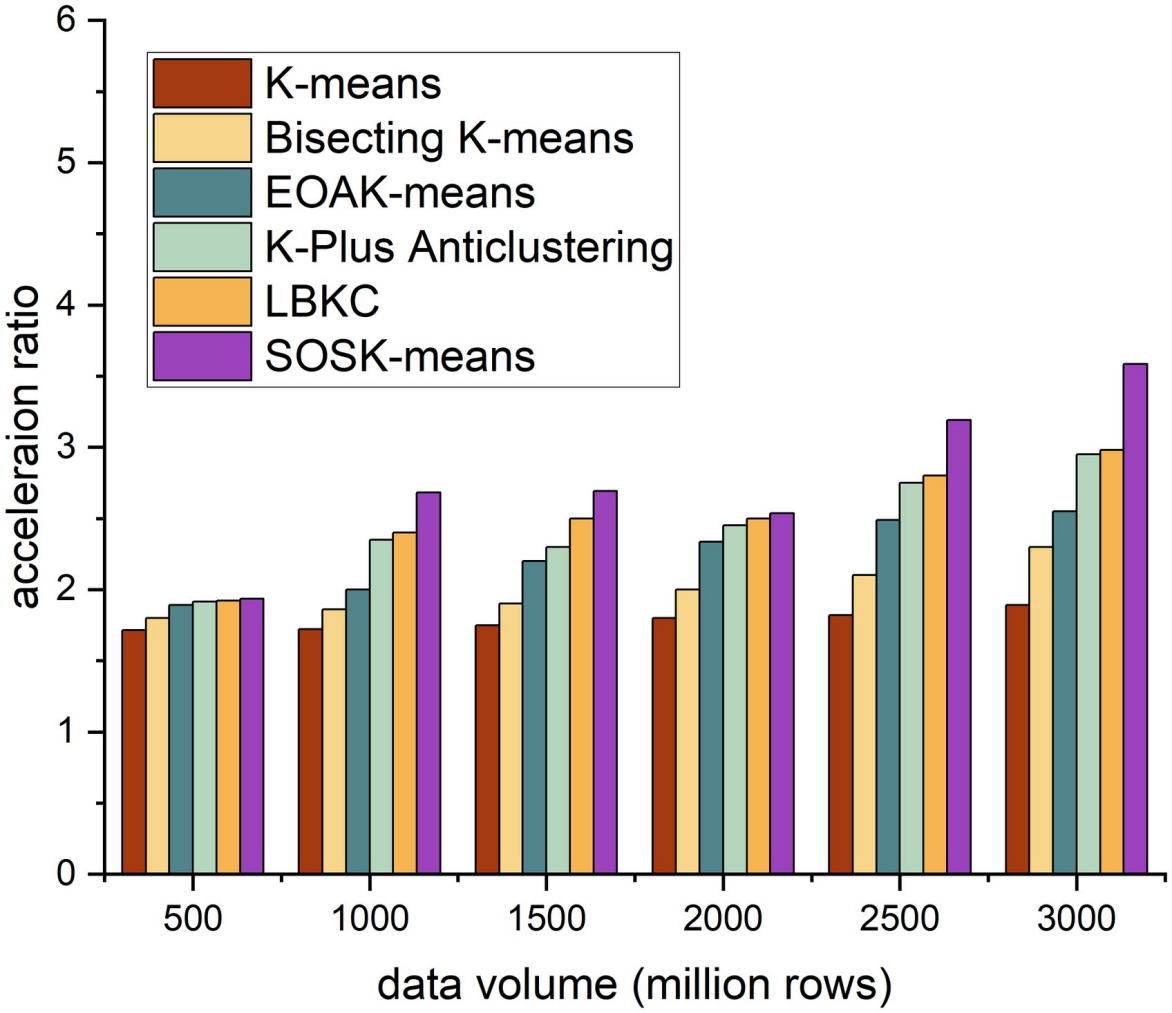
Different algorithms’ performance across 4 cores.

**Fig 3:**
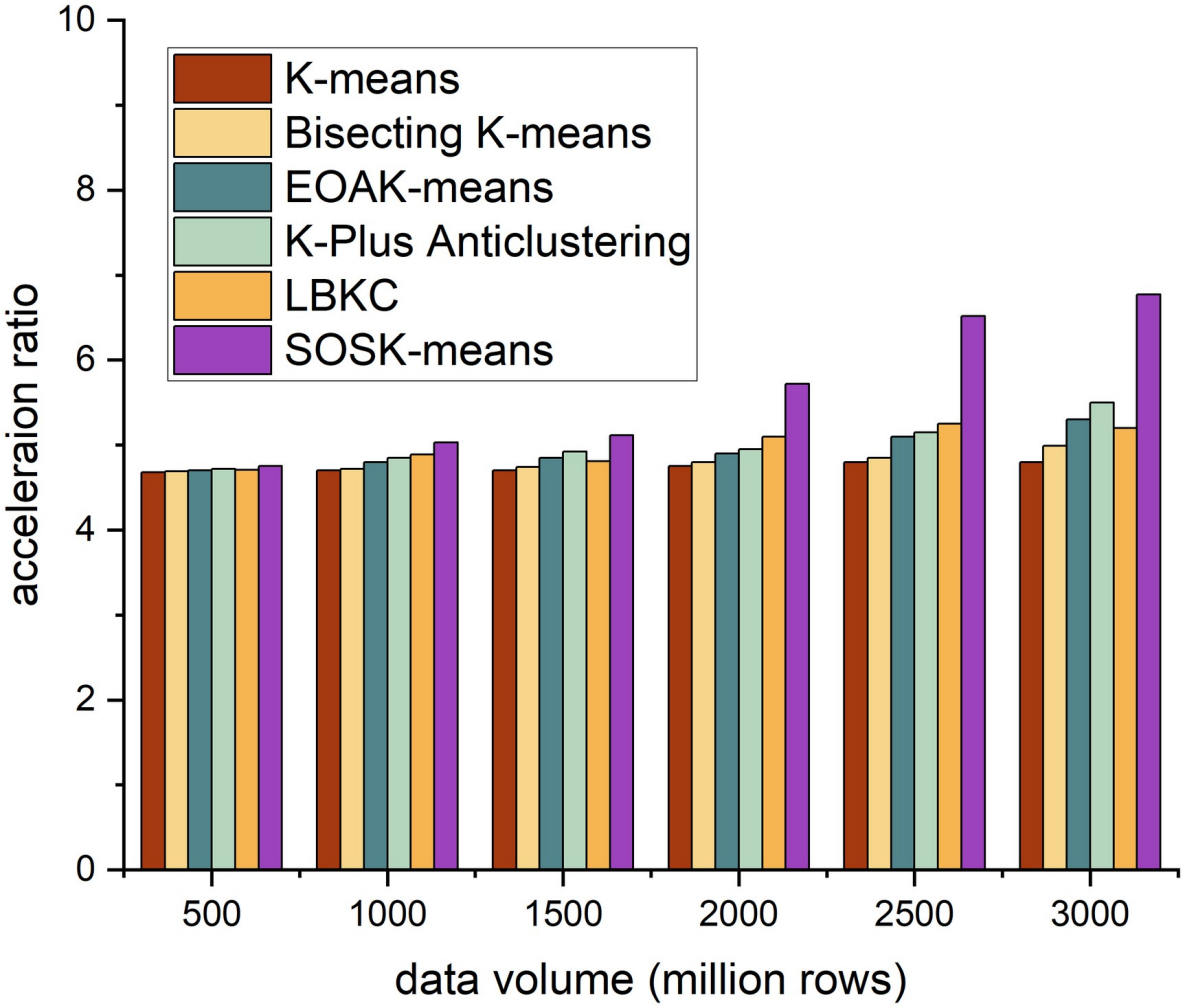
Different algorithms’ performance across 8 cores.

**Fig 4:**
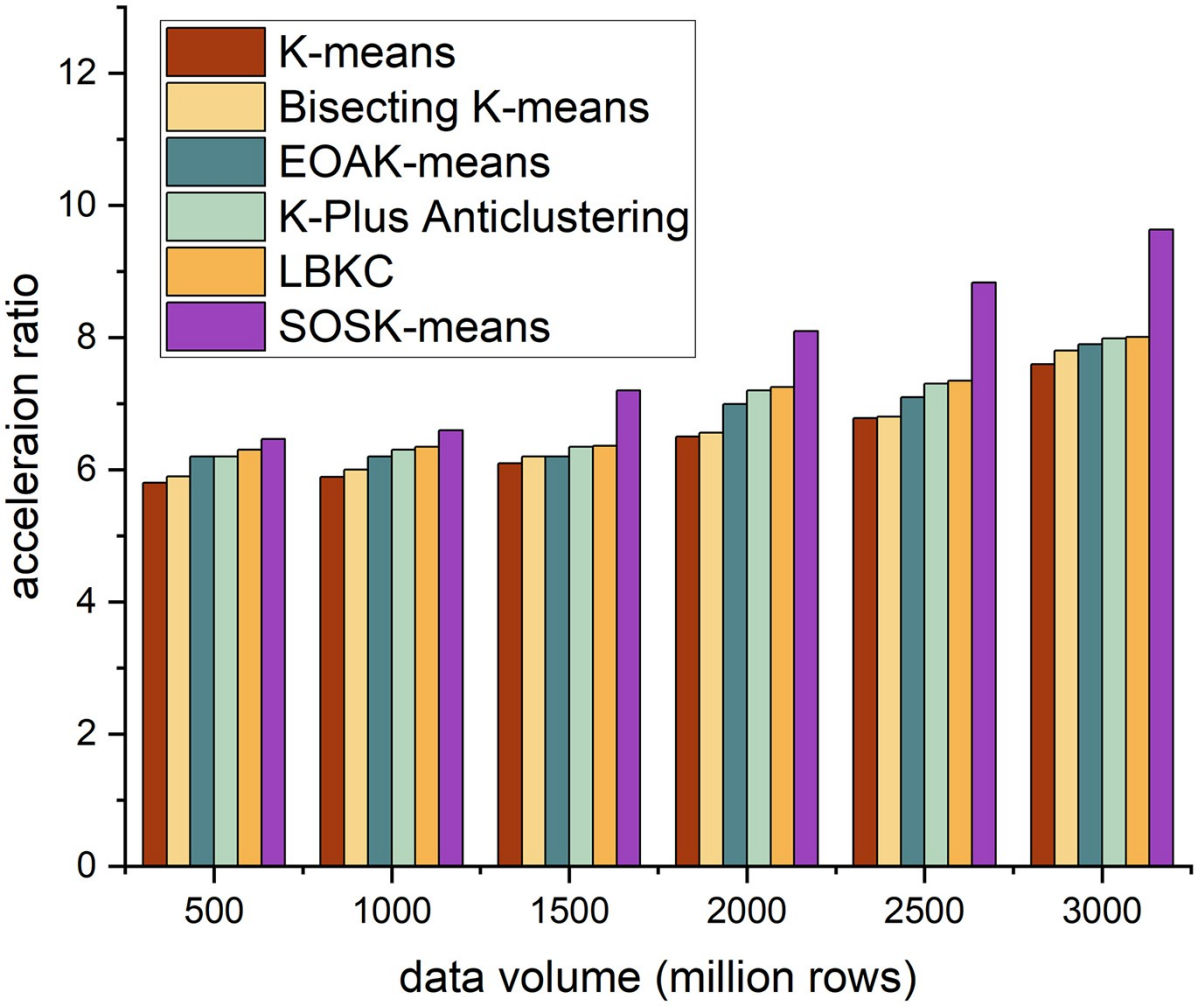
Different algorithms’ performance across 12 cores.

**Fig 5:**
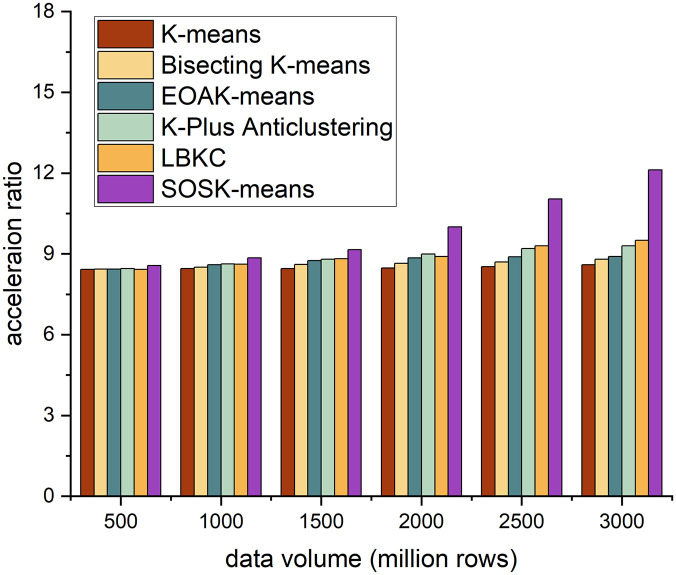
Different algorithms’ performance across 16 cores.

The acceleration ratio is the ratio of the running time consumed by the same task in a uniprocessor system and a parallel processor system. It measures the effect of algorithm parallelization. Figs 2–5 shows the accelerating ratio changes of the four algorithm under different numbers of cores and different data volumes.

On the whole, when the number of cores is constant, as the amount of data increases, the acceleration ratio of the SOSK-Means algorithm shows an increasing trend, and when the number of cores is 4, the acceleration ratio of the algorithm increases slowly. When the data volume is less than 15 million rows, the SOSK-Means accelerating ratio grows slowly because the cluster startup and communication between nodes take considerable time and overhead. When the data volume is greater than 15 million rows, the accelerating ratio approximates a linear increase, and the time to process the data is greater than the cluster deployment time. When the amount of data is constant, the acceleration ratio of the algorithm increases with the increase in the number of cores because an increase in the number of cores means an increase in nodes, which gives play to the time advantage of parallel computing. In general, as the amount of data and the number of cores increase, the acceleration ratio of the SOSK-Means algorithm is larger.

### 5.6 Ablation experiment design

#### 5.6.1 Components

As outlined above, the proposed method improves upon the basic k-means algorithm through five distinct modules. The codes and descriptions of these modules are detailed in Table 8. We evaluated the F1 score and execution time for each selected module and conducted experiments on the CCFD and KDD99 datasets.

**Table 8:** Components details.

Component Code	Description
A	Weighted Jump Library Sampling
B	Weighted Max-Min Distance with Variance
C	Mean Square Error for Initial Center Selection
D	Novel Distance Comparison Method
E	Directed Acyclic Graph (DAG) for Distributed Strategy

#### 5.6.2 Combination ablation experiments

The results are shown in Tables 9 and 10, which indicate that when the A, B, or C components are not used, clustering accuracy decreases. The clustering accuracy gradually improves with the sequential addition of these components. When the D or E components are not used, the efficiency in handling large-scale data significantly decreases. When only the A and B modules are added, clustering accuracy improves noticeably but does not reach optimal levels. When the A and E modules are not added, the efficiency in handling large-scale data significantly decreases.

**Table 9:** Combination ablation experiments on CCFD.

Experiment	A	B	C	D	E	F1-score	time
1	×	×	×	×	×	0.8720	1140.999
2	×	✓	✓	✓	✓	0.8695	1139.426
3	✓	×	✓	✓	✓	0.8706	1138.597
4	✓	✓	×	✓	✓	0.8685	1139.420
5	✓	✓	✓	×	✓	0.8711	1145.861
6	✓	✓	✓	✓	×	0.8708	1141.596
7	✓	✓	×	×	×	0.8621	1141.397
8	✓	✓	×	×	✓	0.8695	1139.381
9	×	✓	✓	✓	×	0.8656	1142.653
10	✓	✓	✓	✓	✓	**0.8803**	**1021.399**

**Table 10:** Combination ablation experiments on KDDc99.

Experiment	A	B	C	D	E	F1-score	time
1	×	×	×	×	×	0.8750	5700.562
2	×	✓	✓	✓	✓	0.8738	5698.596
3	✓	×	✓	✓	✓	0.8736	5698.102
4	✓	✓	×	✓	✓	0.8711	5699.102
5	✓	✓	✓	×	✓	0.8744	5750.982
6	✓	✓	✓	✓	×	0.8689	5705.565
7	✓	✓	×	×	×	0.8703	5703.552
8	✓	✓	×	×	✓	0.8715	5695.782
9	×	✓	✓	✓	×	0.8737	5707.354
10	✓	✓	✓	✓	✓	**0.8816**	**5052.753**

## 6 Conclusions and future works

This paper proposes an improved K-Means algorithm based on the Spark optimization sample. This algorithm uses the weighted max-min distance with variance, which can find distant and dense clusters. Finally, the best initial center is selected by the mean square error. In the iteration, a novel distance comparison method is used to reduce computation time. The algorithm also describes the DAG, which can optimize performance based on distributed strategies. Experimental results show that SOSK-Means significantly improves computational speed while maintaining high computational accuracy.

In response to the current algorithm’s limitations in handling complex shapes and uneven densities in clustering, future research will explore several key directions. First, we plan to enhance the algorithm’s adaptability to complex data structures by integrating advanced clustering techniques, such as density-based methods or neural network approaches. Additionally, we will investigate the application of dynamic weighting schemes to adjust the significance of max-min distance and variance based on real-time data characteristics, thereby improving the algorithm’s flexibility and accuracy.
